# Cannabis medicinal: puntos críticos para su uso clínico

**DOI:** 10.7705/biomedica.6468

**Published:** 2022-09-02

**Authors:** Diego Mauricio Gómez-García, Herney Andrés García-Perdomo

**Affiliations:** 1 Departamento de Medicina Familiar, Escuela de Medicina, Universidad del Valle, Cali, Colombia Universidad del Valle Departamento de Medicina Familiar Escuela de Medicina Universidad del Valle Cali Colombia; 2 Unidad de Urología/Urooncología, Grupo de Investigación UROGIV, Departamento de Cirugía, Escuela de Medicina, Universidad del Valle, Cali, Colombia Universidad del Valle Departamento de Cirugía Escuela de Medicina Universidad del Valle Cali Colombia

**Keywords:** cannabis, cannabinoides, endocannabinoides, eficacia, seguridad, usos terapéuticos, Cannabis, cannabinoids, endocannabinoids, efficacy, safety, therapeutics uses

## Abstract

El cannabis se ha utilizado desde la antigüedad con fines recreativos y medicinales. Es una fuente muy rica de compuestos químicos, la mayoría denominados fitocannabinoides, que tienen una variedad de efectos fisiológicos, principalmente por su unión a receptores cannabinoides endógenos como el CB1 y CB2, entre otros.

El cannabis tiene propiedades terapéuticas potenciales y sus preparaciones se han utilizado como remedios tradicionales para tratar el dolor y la emesis. Los cannabinoides sintéticos se utilizan clínicamente como analgésicos, antiespasmódico, antieméticos y estimulantes del apetito. La toxicidad significativa del cannabis es poco común en los adultos, sin embargo, puede tener múltiples efectos adversos agudos y crónicos.

La calidad de la evidencia en este campo se ha visto limitada por la corta duración de los estudios, los reducidos tamaños de las muestras, la falta de grupos de control y la existencia de sesgos en la mayoría de los estudios revisados. En este contexto, son necesarios más estudios de mejor calidad metodológica para apoyar el uso seguro de esta terapia en otras enfermedades. La decisión de incorporar los cannabinoides como terapia en alguna de las condiciones descritas depende de la evidencia, el uso de terapias previas y el tipo de paciente.

## Abstract (en)

Since ancient times cannabis has been used for recreational and medicinal purposes. It is a significant source of chemical compounds, most of them called phytocannabinoids. These compounds have several physiological effects and produce their effects primarily by binding to endogenous cannabinoid receptors such as CB1 and CB2, among others.

Cannabis has potential therapeutic properties and its preparations have been used as traditional remedies to treat pain and emesis. Synthetic cannabinoids are used clinically as analgesics, antispastics, antiemetics, and appetite stimulants. Significant cannabis toxicity is rare in adults; however, it can produce countless acute and chronic side effects.

The quality of the evidence in this field is limited by the short duration of the trials, poor sample sizes, lack of a control group, and the existence of bias in most of the reviewed studies. Therefore, a larger number of studies with better methodological quality is required to support the safe use of this therapy. The decision to include cannabinoids as a treatment for any of the conditions described will depend on the evidence, the use of previous therapies, and the type of patient.

La necesidad de nuevos tratamientos para el alivio de diversas enfermedades, así como los avances científicos en la comprensión de la biología del sistema endocannabinoide, la farmacología de los cannabinoides, y el desarrollo y la aprobación reguladora de los medicamentos a base de cannabis a nivel internacional (o de sus derivados), han permitido su uso terapéutico. Sin embargo, aún existen controversias sobre su efectividad y seguridad.

Muchos pacientes buscan alternativas a los tratamientos convencionales que no producen alivio o, incluso, traen consigo efectos indeseables. Dicha búsqueda se ve influenciada por la facilidad en el acceso a la información, las redes sociales y el testimonio de pacientes que dicen haber mejorado con su uso. Los productos medicinales con aprobación reguladora deben distinguirse de los llamados aceites o extractos de cannabis, que normalmente se venden en tiendas naturistas, farmacias o por internet, y cuya calidad y composición se desconocen, pues no suelen estar sometidos a los controles más básicos de salubridad y pueden tener contaminantes tanto químicos como biológicos, pesticidas, mohos, levaduras y bacterias ^1^^,^^2^.

En este comentario nos propusimos mostrar la evidencia actualizada sobre la eficacia del cannabis medicinal en el tratamiento de diversas condiciones clínicas.

## Sistema endocannabinoide

El sistema endocannabinoide es un sistema endógeno de señalización que está ampliamente distribuido en el organismo de los mamíferos e interviene en múltiples vías metabólicas que regulan la fisiología celular ^2^. Desempeña un papel clave en la regulación de una gran gama de procesos fisiológicos, incluidos el metabolismo, el estado de ánimo, la función motora, el apetito, el control cardiovascular, la reacción al estrés, el tubo digestivo, las reacciones inmunitaria e inflamatoria, la función endocrina y el dolor.

El sistema endocannabinoide está conformado por distintos elementos: dos receptores principales, CB1 y CB2, pertenecientes a la familia de los receptores acoplados a proteínas G, y otros como el receptor transitorio vaniloide V1 (*Transient Receptor Potential Channel*, TRPV1) o los receptores activados por proliferadores peroxisomales (PPAR). Otros de sus componentes son los ligandos endógenos o endocannabinoides, que son ácidos grasos poliinsaturados derivados del ácido araquidónico, entre los cuales los más importantes son la araquidonil-etanolamida (AEA) y el 2-araquidonilglicerol (2-AG). Además, en el sistema endocannabinoide, se incluyen dos enzimas responsables de la síntesis y degradación de ácidos grasos: la hidrolasa amida de ácidos grasos (*fatty acid amide hydrolase*, FAAH) y la lipasa de monoacilglicéridos (*monoacylglycerol lipase*, MAGL), y también, las vías de señalización intracelular reguladas por los endocannabinoides y sus sistemas de transporte ^3^.

El receptor CB1 se localiza en órganos periféricos y en el sistema nervioso central, y es el más abundante en el cerebro; la mayoría de los receptores del CB1 se encuentran en las terminales axónicas y en los segmentos preterminales del axón, fuera de la zona activa de la sinapsis. La distribución del receptor CB1 está ligada a los efectos farmacológicos que producen los cannabinoides, por ejemplo, en los ganglios basales se correlaciona con la actividad locomotora y, en áreas del hipocampo y corticales, con el aprendizaje, la memoria y la acción anticonvulsiva. Este receptor también está presente a nivel periférico en bazo y amígdalas, corazón, próstata, útero, ovarios, tejido adiposo, músculos, hígado, tubo digestivo y páncreas. El receptor CB2 fue descrito por primera vez en el bazo y, luego, en las amígdalas y en distintas células del sistema inmunitario (linfocitos B, monocitos y linfocitos T); se expresa, además, en corazón, hueso, hígado y páncreas.

Los receptores de cannabinoides CB1 y CB2 están acoplados a proteínas G de tipo inhibidor, cuyas subunidades α inhiben la adenilatociclasa, lo que da lugar a un descenso de los niveles de adenosin monofosfato cíclico (AMPc) intracelular. Ambos fenómenos contribuyen a la reducción de la excitabilidad neuronal y la supresión de la liberación de neurotransmisores ^3^.

## Fitocannabinoides

Los fitocannabinoides son compuestos biológicamente activos presentes en las resinas secretadas a partir de las flores de la planta del género *Cannabis*. Hay más de 120 cannabinoides identificados en la literatura científica. El principal responsable de las propiedades psicotrópicas y psicoactivas de *Cannabis* es el delta 9-tetrahidrocannabinol (THC), el cual tiene una mayor potencia como psicotrópico y actúa como agonista parcial de los dos receptores de cannabinoides (CB1 y CB2). Por otra parte, el cannabidiol (CBD) no tiene propiedades psicotrópicas, tiene efecto antioxidante, interviene en la disminución de los niveles de IL-8, IL-10, TNF-α y IFNµ, y no tiene una actividad agonista apreciable en los receptores CB1, aunque se postula como un modulador alostérico negativo de este.

Otros fitocannabinoides son cannabinol (CBN), cannabicromeno (CBC), cannabiciclol (CBL), cannabigerol (CBG), monometil éter del cannabigerol (CBGM), cannabielsoína (CBE) y cannabinodiol (CBND). Además de los fitocannabinoides, la planta de cannabis también contiene una gran cantidad de terpenos, flavonoides y otros compuestos que, por sí mismos, pueden ser farmacológicamente activos, pero han sido poco estudiados ^1^^,^^3^.

## Usos terapéuticos del cannabis

### Cannabis como estimulante del apetito y antiemético

Es ampliamente sabido que los cannabinoides aumentan el apetito ^2^^,^^4^. Incluso en estado de saciedad, este efecto orexigénico está mediado por la activación del receptor CB1 en las neuronas glutamatérgicas del prosencéfalo, el hipotálamo y en el sistema mesolímbico y su principal neurotransmisor, la dopamina. Funcionan, además, como agentes antieméticos al interactuar con los receptores CB1 y 5-HT3 que se encuentran en el sistema nervioso central (SNC) y en el complejo vagal dorsal (CVD), sitio en el que se activa la emesis.

Son soluciones potentes para las náuseas inducidas por quimioterapia cuando los pacientes no mejoran con los medicamentos tradicionales. Además, son los únicos antieméticos que aumentan el apetito ^1^^,^^5^^,^^6^. El dronabinol, por ejemplo, ha sido evaluado en estudios clínicos a corto y largo plazo para el tratamiento del síndrome de desnutrición y emaciación en pacientes infectados por el HIV ^4^. Se le asoció con el aumento del apetito y la grasa corporal en un 1 %, por la activación de los receptores CB1 en el hipotálamo ^7^.

### Cannabis y enfermedades neurológicas

El efecto antiinflamatorio de los cannabinoides puede ayudar a suprimir la actividad de la esclerosis múltiple, al reducir los factores inflamatorios. Además, cannabinoides como el Sativex® (nabiximol) han sido aprobados en varios países para el tratamiento de la espasticidad grave producida por esta enfermedad, pues actúa en los receptores presinápticos CB1, reduciendo la liberación masiva de glutamato y regulando la excitabilidad glutamatérgica durante la espasticidad ^6^^,^^8^.

Por otro lado, el Epidiolex®, que contiene CBD puro, fue aprobado para formas de epilepsia resistente: el síndrome de Lennox-Gastaut, el síndrome de Dravet y la esclerosis tuberosa ^9^^-^^12^. El mecanismo exacto de la actividad anticonvulsiva del CBD no está del todo claro, aunque, probablemente no se debe al impacto sobre el CB1 o el TRPV1, sino sobre los receptores GPR55, 5-HT1A, FAAH y otras dianas moleculares. En Colombia, su nombre comercial es Neviot® y tiene las mismas indicaciones ^9^^,^^13^.

### Cannabis y cáncer

Aunque hasta la fecha el uso de cannabinoides en el cáncer se limita a aliviar síntomas como las náuseas o el dolor, en algunos estudios se han explorado sus propiedades antitumorales ^14^. El papel del sistema endocannabinoide no está completamente claro en el cáncer, sin embargo, se ha sugerido que los receptores de cannabinoides y los ligandos endógenos se sobreexpresan en el tejido tumoral ^14^. Se ha descubierto que los cannabinoides inhiben la proliferación de células tumorales, la angiogénesis, la invasión tumoral e inducen la apoptosis *in vitro* e *in vivo* mediante la activación de los receptores cannabinoides. No obstante, se requieren más estudios que respalden la idea de introducir los cannabinoides como agentes antitumorales ^15^.

### Cannabis y dolor

La activación de los receptores del sistema endocannabinoide en las neuronas periféricas, espinales y supraespinales, suprime la transmisión nociceptiva. Además, estos receptores están presentes en las células inmunitarias y regulan la reacción inflamatoria ^13^. Los cannabinoides controlan el dolor al actuar sobre varios receptores mediante diferentes mecanismos; por ejemplo, el THC tiene la capacidad de inhibir la síntesis de prostaglandina E-2 y estimular la lipoxigenasa, de disminuir la liberación de 5-hidroxitriptamina (5-HT) en las plaquetas y su captación sinaptosómica, al mismo tiempo que aumenta su producción cerebral con impacto en el sistema trigémino-vascular en las migrañas, en la alteración de la función dopaminérgica, la inhibición de la liberación presináptica de glutamato y la activación del potencial receptor transitorio de los vanilloides-2 (TRPV2) ^16^^,^^17^.

Por otro lado, el CBD puede inhibir o desensibilizar la señalización neuronal del TRPV-1 o agonista del receptor de capsaicina, el cual es capaz de inhibir la enzima FAAH, responsable de la hidrólisis de la anandamida y la inhibición de su recaptación. El CBD tiene la capacidad de inhibir el metabolismo hepático del THC a 11-hidroxi-THC, compuesto este más psicotrópico, y de aumentar su vida media, lo que reduce sus efectos secundarios ^2^. El CBD puede reducir los efectos antiinflamatorios al disminuir las especies reactivas de oxígeno (ROS), los niveles del factor de necrosis tumoral (TNF-α) y las citocinas proinflamatorias. Además, puede inducir la apoptosis de las células T, inhibir su proliferación y reducir la migración y adhesión de las células inmunitarias, lo que resulta en una reducción del estrés oxidativo y la inflamación ^1^^,^^18^.

El dolor crónico, debido al cáncer, neuropático o asociado con la esclerosis múltiple, es un problema importante, especialmente en adultos mayores. En la mayoría de los casos, el dolor crónico se trata con opiáceos, antidepresivos y fármacos anticonvulsivos. Actualmente, los únicos analgésicos para tratar el dolor grave son los opioides, pero estos se asocian con efectos secundarios como sedación, estreñimiento, pérdida de apetito, náuseas, insuficiencia respiratoria, tolerancia, dependencia o riesgo de abuso ^19^. En estos casos, el cannabis se considera una opción contra el dolor y su legalización en algunos países se ha reflejado en una disminución de las muertes por sobredosis debida a opioides. El nabiximol ha demostrado su utilidad en pacientes con cáncer avanzado que requieren dosis de opioides más bajas por intolerancia temprana al tratamiento ^6^^,^^20^.

La *International Association on the Study of Pain* (IASP) señala que los cannabinoides no son efectivos contra el dolor neuropático crónico y no recomienda su uso de manera concluyente. Por otro lado, la *American Academy of Neurology* sugiere que los médicos pueden ofrecer THC o CBD en aerosol bucal o THC oral, para reducir los síntomas de espasticidad y dolor en la esclerosis múltiple ^21^.

En otros estudios no se ha encontrado evidencia de que el consumo de cannabis reduzca la intensidad del dolor o el consumo de opiáceos en personas con dolor crónico no oncológico, dolor osteoarticular o fibromialgia ^22^^,^^23^. Esto resalta la importancia de hacer más pruebas clínicas bien diseñadas en personas con diferentes comorbilidades, para determinar la eficacia del cannabis en el manejo del dolor crónico ^24^.

En el 2017, las *National Academies of Sciences, Engineering and Medicine* de Estados Unidos encargaron a un grupo de expertos hacer una revisión exhaustiva de la literatura existente en torno al efecto del cannabis y los cannabinoides en la salud y el estado actual de la evidencia y las recomendaciones, la cual incluyó más de diez mil artículos. Concluyeron que existe evidencia sustancial de que el cannabis y los cannabinoides son efectivos en el tratamiento de pacientes con esclerosis múltiple aquejados de espasticidad, como tratamiento coadyuvante contra las náuseas y los vómitos inducidos por la quimioterapia, y en el tratamiento de dolor crónico en adultos ^6^.

En la guía publicada por la revista médica BMJ en el 2021, se recomiendan el cannabis medicinal no inhalado y los cannabinoides cuando la terapia estándar no es suficiente para aliviar el dolor. Debe iniciarse con dosis bajas de CBD no inhalado e ir aumentándolas gradualmente, así como la concentración de THC, según la reacción clínica terapéutica y la tolerabilidad, y considerando la experiencia previa con el cannabis y los efectos adversos. Se recomienda iniciar con 5 mg de CBD dos veces al día y aumentar a 10 mg cada dos a tres días, hasta una dosis máxima diaria de 40 mg. Si la reacción cínica terapéutica no es satisfactoria, deben agregarse 1 a 2,5 mg de THC por día y ajustar con 1 a 2,5 mg cada dos a siete días, hasta un máximo de 40 mg/día ^25^ (cuadro 1).

**Cuadro 1:** Cannabinoides utilizados para el tratamiento del dolor

Cannabinoide sintético	Contenido	Indicación	Dosis
Marinol^™^ (dronabinol) y Cesamet^™^ (nabilona)	Forma oral sintética de THC y agonista parcial de los receptores CB1 y CB2. Aprobado en los Estados Unidos en 1985 para las náuseas asociadas con la quimioterapia y para la estimulación del apetito en el VIH/sida	Dolor crónico en pacientes con esclerosis múltiple, náuseas y vómitos asociados con la quimioterapia contra el cáncer y como estimulante del apetito en el VIH/ sida.	2,5 a 40 mg al día
Cesamet^™^ (nabilona)	Análogo del dimetilheptílico sintético del THC administrado por vía oral. Aprobado en 1981 por la FDA de Estados Unidos para el tratamiento de las náuseas y los vómitos inducidos por la quimioterapia	Náuseas y vómitos inducidos por quimioterapia. En algunos estudios se ha probado por fuera de las indicaciones para el tratamiento del dolor crónico, de la fibromialgia y como estimulante del apetito en VIH/sida	0,2 a 6 mg al día
Nabiximols (Sativex^™^) aerosol oromucosal	*Spray* bucal a base de cannabis que contiene una mezcla de una proporción 1: 1 de THC y CBD. Aprobado en varios países europeos	Se utiliza como tratamiento complementario contra el dolor neuropático y la espasticidad relacionada con la esclerosis múltiple en pacientes que no mejoran con los tratamientos antiespásticos convencionales.	16 aspersiones orales al día

## Seguridad del cannabis

Hay varios efectos no deseados que pueden asociarse con el consumo de cannabis y cannabinoides sintéticos. La exposición a altas concentraciones de THC podría provocar efectos psicológicos (euforia, delirios, ansiedad, ataques de pánico) y neurológicos (mareos, somnolencia, ataxia, convulsiones, hipotonía, estupor, coma o alteraciones oculares como midriasis e hiperemia conjuntival), además de trastornos gastrointestinales y efectos cardiovasculares (taquicardia, hipertensión arterial sistémica e hipotensión postural) ^26^^,^^27^.

Estos efectos secundarios pueden atribuirse al bajo o nulo contenido de CBD, el cual tiene un papel protector (propiedades ansiolíticas y antipsicóticas), o a la gran afinidad del THC por los receptores CB1 ^1^. Los efectos adversos inducidos por el cannabis pueden verse influidos por otros factores como la variación genética, la edad, el sexo, la etnia, y la duración y frecuencia del consumo de cannabis ^1^. Los efectos adversos del THC, como fatiga, taquicardia y mareos, se evitan comenzando con dosis bajas y titulando lentamente las preparaciones de cannabis. Además, la combinación de CBD con THC puede equilibrar los efectos secundarios de este último ^25^.

En el sistema cardiovascular, el cannabis puede inducir aumento de la frecuencia cardíaca y la presión arterial, así como vasodilatación sistémica e hipotensión ortostática; además, puede desencadenar arritmias, miocarditis e infartos de miocardio en pacientes con enfermedad cardiovascular descompensada debido a la estimulación de los receptores CB1. Generalmente, los síntomas cardiovasculares inducidos por el cannabis son bien tolerados por la mayoría de las personas jóvenes sanas, pero puede no ser así en los pacientes con enfermedades coronarias establecidas ^28^^,^^29^.

En el sistema nervioso, la activación de los receptores CB1 puede provocar efectos secundarios centrales, como ataxia y catalepsia. La unión del THC a dichos receptores puede afectar la percepción, la memoria y el movimiento como resultado de la inhibición selectiva de la actividad del adenilato ciclasa a consecuencia de la activación de los CB1; además, puede causar disforia y tener efectos psicomiméticos ^30^. El consumo de grandes cantidades de THC está relacionado con el deterioro cognitivo y de la memoria de corto plazo, cuya aparición después del consumo de cannabis se atribuye al efecto del THC sobre los receptores CB1 del hipocampo. Los agonistas de THC y CB1 reducen la liberación de neurotransmisores presinápticos y, en consecuencia, alteran la plasticidad sináptica a largo plazo en el hipocampo. Aunque en comparación con el consumo de alcohol, el consumo prolongado de cannabis no se asocia con un deterioro cognitivo grave, sí hay problemas de memoria relacionados con el consumo de cannabis a corto y largo plazo, los cuales aparecen como un deterioro sutil de la memoria y la atención, así como de la capacidad para integrar y organizar información compleja ^1^^,^^31^.

Otros efectos centrales del cannabis incluyen la alteración del rendimiento psicomotor, la función motora y el tiempo de reacción. De hecho, la administración aguda de cannabis se asocia con una etapa inicial de excitación y agitación psicomotora, seguida de un estado de disartria, ataxia, inercia física y descoordinación general. Esto hace que el cannabis genere un riesgo importante de accidentes de tránsito y de violencia interpersonal ^32^. Los pacientes deben evitar conducir o utilizar maquinaria cuando inician el uso de cannabis medicinal o mientras se ajustan las dosis ^25^.

En general, el efecto psiquiátrico adverso más común con el uso del cannabis es la ansiedad ^26^^,^^33^. No obstante, se encontró una relación entre su consumo y el riesgo de psicosis tóxica aguda y trastornos del espectro esquizofreniforme ^34^. El impacto del consumo de cannabis puede diferir en personas con trastornos psiquiátricos. Existe un factor de riesgo significativo de intento de suicidio en pacientes psiquiátricos, en desempleados y en personas con trastornos del estado de ánimo. Las dosis bajas de THC (2,5 a 15 mg) pueden afectar la memoria, la función motora y tener otros efectos secundarios agudos. Por otra parte, las dosis elevadas (>100 mg) pueden producir efectos adversos crónicos como delirios, desorientación, alucinaciones visuales y auditivas, ideas paranoicas, manía y psicosis esquizofreniforme, reacciones que suelen estar relacionadas con la dosis y son autolimitadas ^35^^,^^36^. Los consumidores de cannabis con antecedentes familiares de trastornos psicóticos son particularmente vulnerables ante estos efectos. Existe evidencia suficiente de que el uso frecuente de cannabis por parte de los jóvenes podría aumentar el riesgo de desarrollar trastornos psicóticos o de presentar un primer episodio prematuro de psicosis. También, se ha descubierto que el cannabis agrava enfermedades mentales preexistentes ^37^^,^^38^.

## Análisis del contexto de aplicación y desarrollo de cannabinoides en Colombia

Es importante resaltar que el uso medicinal adecuado del cannabis medicinal en Colombia o en otros países latinoamericanos depende de múltiples factores: voluntad política, aceptación médica, acceso para los pacientes e inversión de la industria. Aunque hay voluntad política y se han emitido múltiples normas que han permitido algunos avances en este campo (Decreto 2467 de 2015, Decreto 780 de 2016, Decreto 613 de 2107 y Decreto 631 de 2018), quedan pendientes diversos aspectos de su administración, y es fundamental darle vía a los procesos reguladores existentes para aprovechar el mundo de posibilidades que hoy se abre en este sentido ^39^^,^^40^ (cuadro 2).

**Cuadro 2:** Tipos de licencias en Colombia, modalidad e institución otorgante ^40^

Tipo de licencia	Modalidad	Institución otorgante
Licencia de fabricacion de derivados de cannabis	Para uso nacional Para investigacion cientifica Para exportacion	Instituto Nacional de Vigilancia de Medicamentos y Alimentos - INVIMA
Licencia de cultivo de cannabis psicoactivo	Para produccion de semillas para siembra Para produccion de grano Para fabricacion de derivados Para fines cientificos Para almacenamiento Para disposicion final	Ministerio de Justicia y del Derecho
Licencia de cultivo de cannabis no psicoactivo	Para produccion de grano y de semillas para siembra Para fabricacion de derivados Para fines industriales Para fines cientificos Para almacenamiento Para disposicion final	Ministerio de Justicia y del Derecho
Licencia de semilla	Para comercializacion o entrega Para fines cientificos	Ministerio de Justicia y del Derecho

Como queda dicho, son bien conocidos los beneficios del cannabis medicinal; sin embargo, aún no se acepta totalmente su uso en el gremio de profesionales de la salud. Sería importante desarrollar campañas de concienciación sobre las áreas específicas de su uso para que se genere mayor demanda y se logre un avance en el desarrollo de esta área llena de oportunidades.

## Conclusiones

El cannabis tiene propiedades terapéuticas potenciales, y sus preparaciones se han utilizado como remedios tradicionales para tratar el dolor y la emesis. Los cannabinoides sintéticos se utilizan clínicamente como analgésicos, antiespásticos, antieméticos y estimulantes del apetito. La toxicidad significativa del cannabis es poco común en los adultos, pero puede causar una amplia gama de efectos adversos agudos y en el largo plazo, dependiendo de la dosis.

La calidad de la evidencia en torno al uso de medicamentos a base de cannabis para el dolor se ha visto limitada por la corta duración de los estudios, los reducidos tamaños de muestra, la ausencia de grupo de control y la existencia de sesgos en la mayoría de los estudios revisados. La decisión de incorporar los cannabinoides como analgésicos dependerá de la gravedad del dolor subyacente y del resultado de otros tratamientos probados que hayan fracasado, y deben considerarse solo como terapia de tercera línea en pacientes seleccionados.
